# Motivations for Undertaking DNA Sequencing-Based Non-Invasive Prenatal Testing for Fetal Aneuploidy: A Qualitative Study with Early Adopter Patients in Hong Kong

**DOI:** 10.1371/journal.pone.0081794

**Published:** 2013-11-27

**Authors:** Huso Yi, Nina Hallowell, Sian Griffiths, Tak Yeung Leung

**Affiliations:** 1 Health Ethics and Medical Humanities Unit, Centre for Global Health, JC School of Public Health and Primary Care, The Chinese University of Hong Kong, Shatin, New Territories, Hong Kong SAR, China; 2 PHG Foundation, Cambridge, Cambridgeshire, United Kingdom; 3 Department of Obstetrics and Gynecology, Faculty of Medicine, The Chinese University of Hong Kong, Shatin, New Territories, Hong Kong SAR, China; Alberta Provincial Laboratory for Public Health/ University of Alberta, Canada

## Abstract

**Background:**

A newly introduced cell-free fetal DNA sequencing based non-invasive prenatal testing (DNA-NIPT) detects Down syndrome with sensitivity of 99% at early gestational stage without risk of miscarriage. Attention has been given to its public health implications; little is known from consumer perspectives. This qualitative study aimed to explore women’s motivations for using, and perceptions of, DNA-NIPT in Hong Kong.

**Methods and Findings:**

In-depth interviews were conducted with 45 women who had undertaken DNA-NIPT recruited by purposive sampling based on socio-demographic and clinical characteristics. The sample included 31 women identified as high-risk from serum and ultrasound based Down syndrome screening (SU-DSS). Thematic narrative analysis examined informed-decision making of the test and identified the benefits and needs. Women outlined a number of reasons for accessing DNA-NIPT: reducing the uncertainty associated with risk probability-based results from SU-DSS, undertaking DNA-NIPT as a comprehensive measure to counteract risk from childbearing especially at advanced age, perceived predictive accuracy and absence of risk of harm to fetus. Accounts of women deemed high-risk or not high-risk are distinctive in a number of respects. High-risk women accessed DNA-NIPT to get a clearer idea of their risk. This group perceived SU-DSS as an unnecessary and confusing procedure because of its varying, protocol-dependent detection rates. Those women not deemed high-risk, in contrast, undertook DNA-NIPT for psychological assurance and to reduce anxiety even after receiving the negative result from SU-DSS.

**Conclusions:**

DNA-NIPT was regarded positively by women who chose this method of screening over the routine, less expensive testing options. Given its perceived utility, health providers need to consider whether DNA-NIPT should be offered as part of universal routine care to women at high-risk for fetal aneuploidy. If this is the case, then further development of guidelines and quality assurance will be needed to provide a service suited to patients’ needs.

## Introduction

The discovery of cell-free fetal DNA (cff-DNA) in maternal blood during pregnancy has created a paradigm shift in the practice of obstetric care, introducing the possibility of non-invasive prenatal testing (DNA-NIPT) [1-3]. Research has shown the clinical validity of non-invasive prenatal tests based on cff-DNA to detect fetal aneuploidies, including trisomy 21 (Down syndrome), 13 (Patau syndrome) and 18 (Edwards syndrome), as early as 10 weeks of gestation with sensitivity and specificity of over 99% [4-7]. DNA-NIPT, using a “massively parallel sequencing” method [8], has been available as a self-paid referral service in the private sectors in Hong Kong (HK) since December 2011. The test is also available in the US. The International Society for Prenatal Diagnosis (ISPD) and the American College of Obstetricians and Gynecologists (ACOG) support the application of DNA-NIPT among high-risk women as part of a package of tests including other risk assessments [9,10]. These ISPD and ACOG guidelines define several categories of women as high-risk, namely, women aged 35 and older, women whose risk scores were found to be high by the conventional serum and nuchal translucency (NT) ultrasound based Down syndrome screening (SU-DSS) tests [11], and women with personal or family history of fetal aneuploidies. It is likely that DNA-NIPT may eventually supplement existing prenatal screening services, particularly for patients at increased risk of aneuploidy. 

The clinical benefits of DNA-NIPT include a reduction in the use of invasive prenatal diagnosis tests (IPD), such as chorionic villus sampling (CVS; usually performed at 10-13 weeks of gestation) and amniocentesis (performed at 15-20 weeks), which carry about a 0.5-1% procedural risk of miscarriage [12,13], and are carried out in response to relatively high false positive rates from SU-DSS [14,15]. As a consequence, DNA-NIPT could potentially reduce both direct and indirect costs of IPD among high-risk women [16]. Finally, DNA-NIPT can be undertaken during the first trimester and there is evidence that gaining information about aneuploidy early in pregnancy can reduce anxiety among pregnant women and allow them to make better-informed choices regarding the need to undergo further invasive diagnostic procedures, which may, in turn, result in the decision to terminate the pregnancy (TOP) [17,18].

There has been increasing discussion about the ethical, legal, and social implications (ELSI) of the use of DNA-NIPT. Discussion focuses on issues such as: patient autonomy, informed choice, consent procedures, genetic counseling, rights of unborn children (e.g., in the context of TOP), non-clinical application of DNA-NIPT (e.g., sex selection and paternity testing), government regulation and social-cultural values of disabilities [19-26]. Prior to widespread implementation of this technology, it is, therefore, important to undertake research on users’ views of DNA-NIPT, particularly women’s motivations for undertaking DNA-NIPT [18,27-29]. There is little published data on the views and experiences of pregnant women who have undertaken DNA-NIPT in contexts where the test is commercially available [29]. The literature has discussed the potential clinical application of DNA-NIPT, but only before it was offered clinically and these studies explored how the test should meet the standards prescribed by a national healthcare system [26,30-33], or patients’ preference of being informed about aneuploidy using DNA-NIPT [34]. This paper reports research that investigated women’s motivations for undertaking DNA-NIPT to screen for Down syndrome and their perceptions of the testing process using a qualitative narrative analysis method. (Note: For the DNA-NIPT of “safeT21; Sensitive Analysis of FoEtal DNA for Trisomy 21 screening,” the plasma harvesting steps were performed in HK while the subsequent steps were performed by a commercial laboratory in the USA.)

## Methods

### Research Context

In July 2010, HK Hospital Authority instituted a universal screening program under which pregnant women, irrespective of their age, are offered either the first trimester combined SU-DSS or the second trimester double test (i.e. blood test on level of alpha-fetoprotein and total human chorionic gonadotropin) in the public hospital [35]. These tests are also available in private hospitals or laboratories and typically cost between HK$ 2,000-3,000 (US$ 260-390) for SU-DSS (e.g., OSCAR; One-Stop Clinic for Assessment of Risk) [15,36] and between HK$ 4,500-7,500 (US$ 580-970) for IPD. The criteria of the risk assessment from SU-DSS are different in the public and private services. A risk ratio of higher than 1 in 250 is considered as positive or high-risk in the public sector while it varies in the private sector. Women who are screened as “high-risk” are given the option of CVS or amniocentesis. If they are prepared to pay for the test, women can undergo faster tests such as quantitative fluorescent polymerase chain reaction (PCR).

### Sampling and Recruitment

Women were recruited between June and December 2012 from a university-affiliated private clinic in HK that offers DNA-NIPT. A private clinic was chosen because the test is only available in private, although referrals for NIPT can be made by practitioners in the public sector. A study information pack, including study details and consent form was distributed to all patients (N=155) while they waited for the test, and 107 women (69%), who agreed to be approached for interview, left their contact information on the form. Of the 48 women who refused to participate in the interview, 23 (47.9%) were at high-risk for Down syndrome according to their referral form. There was no sampling bias by SU-DSS risk status. The recruitment was conducted independently from the clinic service so that the study was not regarded as a part of the service. To achieve maximum variability in the sample, purposive sampling methods were used [37]. A quota-sampling matrix was constructed using women’s age, risk ratio (i.e., high-risk of 1 in 250) and gestational age, in addition to residence type from the contact information (e.g., government subsidized housing and private estate was used as a proxy of socioeconomic status) to systematically recruit a diverse sample in terms of risk factors for Down syndrome, timely accessibility, and financial affordability. The income limit for a family of two persons for government subsidized housing is HK$ 13,750 (≈US$ 1,770). It is generally assumed that women who reside in government subsidized housing have lower income level and poor socio-economic status in comparison to those who reside in private housing. Women were contacted prior to receiving their result and interviewed during the week after they received the result. 

Using theoretical sampling, we identified emerging themes from the early and intermediate stages of interviews and recruited women who would be represented by the themes in an attempt to reach theoretical saturation (i.e. no new themes emerged in the interviews and pre-identified themes were recurrent) [38]. After completing each interview, the interviewer wrote an interview note within a day, which contained an overall description of the interview setting, particular points (e.g., questions and responses) to be noted, questions to be revised or further explored, and self-reflections on interview. The interview transcripts were completed within three days after interview. Every week, the data analysis meeting was held to discuss interview notes and review the transcripts to evaluate the quality of interview data. During the data collection phase we conducted observation and noted informal interviews with obstetricians and midwives in the study clinic. We also collected materials about DNA-NIPT from the websites of medical providers and consumers (e.g., pregnant women), YouTube, newspapers, and magazines. Triangulating the interview notes and ethnographic data, we identified the following themes: self-referral as direct-to-consumer approach (i.e., those who made appointment directly to the study clinic without referral), previous experience with TOP due to fetal abnormalities and miscarriage, low income (i.e., monthly household income is similar to the testing fee of HK$8,000; US$1,000), and twin pregnancy as the advance of DNA sequencing technology enabled to detect cff-DNA of twins. These emerging themes guided our recruitment strategy in the later stages of the project. Theoretical saturation was reached after 45 interviews. 

### In-depth interview

Since the topics of this study are under-researched and highly sensitive and the study aim was to explore individual patients’ subjective experiences of informed decision-making and consent for DNA-NIPT a qualitative in-depth interview approach was deemed the most appropriate methodology. A topic guide was developed to help interviewer and interviewees elaborate on their experiences. The final guide was reviewed by obstetricians and midwives, and pilot tested with three patients to ensure the content was appropriate; the pilot interview data were not included in analysis. Semi-structured conversational interviews were undertaken. Women were asked to comment on the following: decision-making about undertaking DNA-NIPT, information needs, information sources, experiences of antenatal screening, the informed consent process, discussion of the test result with healthcare professionals and family. The participants provided their written consent to participate in the study. All interviews were audio-taped with consent. Since the interview contained information of a sensitive nature, demonstrating empathy and genuine interest with consideration for participants’ situation was crucial to collect quality data. An anthropologist who was a mother in her late 30s conducted the interviews. All the interviews took place either in the research office in the school of public health or at the interviewee’s home. No interview was done in the clinic in order to avoid being seen as carrying out “clinical-related research” by the participants. No one was present during the interview besides the participant and interviewer to protect interviewees’ privacy. The average interview length was one and a half hours (range 1-2.5 hours). The average time interval between the women’s undertaking DNA-NIPT and interview was 34 days (median 32 days, SD = 7.8 days, range: 20 to 48 days). This interval allowed the participants to recollect their experience and to potentially reduce recollection-related bias. Upon the completion of the interview, participants received a voucher of HK$ 1,000 (≈US$ 125). The research protocol, including the study information, consent procedure and interview questions, was approved by the Chinese University of Hong Kong – Hospital Authority New Territories East Cluster Clinical Research Ethics Committee and the Survey and Behavioral Research Ethics Committee.

### Data analysis

Audio recordings of all interviews were transcribed verbatim, translated in to English, if necessary, and entered into a QSR NVivo qualitative data analysis software [39]. Back-translation was undertaken to ensure the reliability of translations [40]. NVivo enabled cross-linking and ranking of domains, sub-domains, and relevant transcript quotes. The resulting network of thematic nodes enabled identification of overarching domains. Data analysis was based upon phenomenological analysis [41]. This narrative-based analytical approach pays attention to a patient’s subjective experiences and the meanings contained in these experiences [42], focuses on explanations given (for example, decision-making about DNA-NIPT) [43], and it requires, or acknowledges, the existence of prior knowledge about a research topic [44]. The first author, who is an expert on qualitative health research, completed descriptive and analytic coding of the data with representative quotes, which were further refined through member-checking by a subset of research collaborators, including medical anthropologist with her expertise in genetic testing and bioethics (NH), obstetrician (TYL), public health policy expert (SG), and the genetic scientists (YMDL and RWKC) who developed and implemented DNA-NIPT in HK. Throughout this process, we ensured inter-reliability of the coding. The potential utility of DNA-NIPT – easy, safe, accurate and early detection – has been discussed in the existing ELSI literature and this was used to inform a priori coding for the initial stages of analysis. However, this inductive methodology is based upon themes emerging from the patients’ narratives, hence the final analysis is grounded within the data and reflects their experiences [38,45]. Finally, to acknowledge the influence of interviewee - interviewer dynamics on the data collected, the first author and interviewer held regular data analysis meetings in which we discussed the interviewer’s personal reflections on the interviews, and her assumptions and preconceptions concerning genetic testing, termination of pregnancy, disability, parenting, and family. We would argue that this reflexive exercise enhanced the credibility of the data in the final analysis. In reporting the interview data we will focus upon women’s information sources, referral routes, motivations for testing.

## Results

### Characteristics of interviewees

Forty-five women were recruited and interviewed. Table 1 shows the characteristics of the participants. Their mean age was 35.7 years ranging from 27 to 44 years old and 51.1% were ≥ 35 years old. With respect to their risks, 31 (68.9%) had SU-DSS with risk scores < 1:250), 4 were at increased risk because of their age (≥ 35 years), 2 reported trisomy 21 during a previous pregnancy, and 8 had none of these risk factors. The mean weeks of gestation was 13.5 ranging from 11 to 19 weeks and 77.8% underwent DNA-NIPT by the 14th week. The household income of the majority (88.9%) was above the median for HK (HK$ 20,500 per month ≈ US$ 2,600) as recorded in the 2011 census. About two-thirds (66.7%) had completed university education and 82.2% had a regular job. Ten of the 45 women interviewed (22%) lived in public housing; 4 of these women reported their monthly income under HK$10,000 and 3 reported HK$ 10,000-20,000. In comparison, of the 35 women living in private housing, there was only 1 woman from each of the two income categories (χ^2^ = 22.4, *p* < .001). The residence type was also related to educational attainment; the private housing women were more highly educated. Nine women in public housing only completed high school compared with 3 (8.6%) women in private housing. Eighteen (51.4%) women from private housing had an associate or bachelor degree and 14 (40%) had a master’s degree or above (χ^2^ = 26.6, *p* < .001). There was no relationship between age and housing. Overall, the resident type was found to be a useful indicator to screen for women with low-SES status before interview.

**Table 1:** Characteristics of interviewees (N=45).

	N	%
Age (range 27-44)	35.7 (mean)	3.7 (SD)
25-30	4	8.9
31-34	18	40.0
35-40	19	42.2
41-45	4	8.9
Gestation Weeks (range 11-19)	13.5 (mean)	1.6 (SD)
11	6	13.3
12	3	6.7
13	16	35.6
14	10	22.2
15	6	13.3
16	2	4.4
17	1	2.2
19	1	2.2
Risk Indicators		
Positive result from screening	31	68.9
Ratio < 1:100	10/31	32.3
Ratio 1:101 – 1:250	21/31	67.7
Age ≥ 35 alone without positive screening	4	8.9
Previous pregnancy with trisomy 21	2	4.4
Education		
High School	12	26.7
Associate or Bachelor	19	42.3
Master	14	31.1
Occupation		
Professionals	16	35.6
Manager	7	15.6
Clerk	14	31.1
Housewife	8	17.8
Household Income *		
HK$10,000-HK$19,999	5	11.1
HK$20,000-HK$29,999	4	8.9
HK$30,000-HK$39,999	9	20.0
HK$40,000-HK$49,999	5	11.1
>HK$50,000	22	48.9
Religion		
Christian	9	20.0
Buddhist	2	4.4
None	34	75.6

* HK$ 10,000 ≈ US$1,300

Table 2 shows various milestones in the DNA-NIPT pathway. There were roughly two two-week intervals between undertaking SU-DSS and DNA-NIPT, and receiving the DNA-NIPT result: The mean time at which SU-DSS occurred was 11.9 weeks (*SD* = 1.7, range: 5-17), this was followed by DNA-NIPT at a mean of 13.4 weeks (*SD* = 2.1, range: 10-19), and receiving the result at a mean time of 15.5 weeks (*SD* = 1.9, range: 11-20). Nine women learned about, and decided to undertake, DNA-NIPT before their first screening consultation with obstetric professionals. This group said they had asked their obstetricians for DNA-NIPT at their first appointment. Due to the small sample, statistical inference is not appropriate; however, the women in this group tended to be more educated (78% over bachelor level vs. 56%) and higher earners (67% monthly income over HK$ 50,000 vs. 40%) than those who chose DNA-NIPT after discussing with the obstetric professionals. 

**Table 2:** Milestones of clinical pathway of DNA-NIPT for Down syndrome (weeks of gestation).

Event	N/A*		M (SD)		Median		Range	
Aware of pregnancy	0		5.1 (1.6)		5		3-10	
First visit to prenatal clinic	0		7.0 (2.3)		7		4-14	
First screening for Down syndrome	2		11.9 (1.7)		12		5-17	
First talk about DNA-NIPT with doctors	6		11.7 (3.4)		13		2-19	
First talk about DNA-NIPT with nurses	9		12.9 (2.1)		13		8-19	
First talk about DNA-NIPT with family or friends	1		12.0 (3.0)		13		4-18	
First consultation on DNA-NIPT in clinics	8		12.9 (2.7)		13		5-19	
Decide to take DNA-NIPT (One woman learned and decided to undertake it before pregnancy)	0		12.2 (2.6)		13		5-17	
Undertake DNA-NIPT	0		13.5 (2.0)		13		10-19	
Discuss DNA-NIPT with family or friends	4		15.1 (2.0)		15		11-20	
Receive the test result	0		15.9 (2.0)		16		11-21	
Consultation on the test result with doctors	25		16.3 (2.0)		16		13-21	
Talk about the test result with family or friends	3		15.6 (1.7)		16		11-21	
	N		%					
Decide to undertake DNA-NIPT before									
First consultation with DNA-NIPT	9		20.0						

Note. * The number of women who did not engage in the event.

### Information sources about DNA-NIPT

An examination of women’s information sources about the newly introduced DNA-NIPT is critical to any discussion of its clinical utility and adoption. Many of our interviewees said they had obtained most of their knowledge about DNA-NIPT from the media, including consumer websites (e.g. pregnant women forums), news media, professionals (e.g. online medical forums) and academic institutions. Given that the university had issued a press release about the test in April 2012, information about the university-based test was widely available in the media. Indeed, all the major newspapers in HK had covered the launch of the test addressing the following issues: (1) academic information about the university and department, (2) the rigor of the research underlying the test (e.g., fifteen years of research on clinical discovery and validity), (3) test availability (e.g., HK and US), (4) the nature of the procedure (e.g., DNA sequencing to detect Down syndrome by directly analyzing a blood sample from the mother), (5) test accuracy (e.g., 99.1% sensitivity and 0.1% false positive rate), and (6) potential utility of the test (e.g., possible alternative to IPD that is associated with a miscarriage rate of 0.5% to 1%). 

The emphasis upon the academic credentials of researchers involved in developing the test and the university hospital where the test could be accessed in these media reports appeared to be an important influence on women’s motivations to access testing. Women talked about the importance of having the test in a trusted institution with a good reputation.

*I trust the university and many people say the university hospital prenatal testing is convincing and its accuracy was higher than other hospitals. That’s why even though people are not going to give birth over there, they still book for prenatal testing.*

Although consumer websites were seen as presenting a positive view of DNA-NIPT technology, many women questioned their trustworthiness. Yet, as the next section demonstrates, the existence of easily accessible information sources about new technologies can be seen as important not least, because a minority of women commented that they felt that their health care professionals were not well informed about DNA-NIPT.

### Factors influencing decision-making about DNA-NIPT

Women provided a number of reasons for undertaking DNA-NIPT including: to reduce uncertainty about SU-DSS results, to obtain reassurance about risk and further insurance, to access an easy and safe procedure for women and fetuses, to detect a fetal condition early in pregnancy, and perceived cost-benefits. We report age and SU-DSS risk score in the quotes from high-risk women. 

### Patients self-initiating the discussion about DNA-NIPT

While most of the women in our study opted for DNA-NIPT after their obstetricians offered the option when discussing the results of SU-DSS, nine women initiated the discussion about DNA-NIPT at their first visit to their own private obstetricians.

*I knew about DNA-NIPT before pregnancy and I immediately asked my obstetrician for a referral letter at five weeks’ gestation. If I did not know about this test, my doctor would not have recommended it.*

Another group of women self-initiated the discussion about DNA-NIPT after receiving the result of SU-DSS.

*My doctor only mentioned amniocentesis or CVS. No mention about DNA-NIPT. I had to find information for myself and made a self-referral for the test. (33 years old, 1:133*)

These women commented that they had initiated the discussion about DNA-NIPT because they were of the opinion that their obstetricians had little knowledge about the test or how to access DNA-NIPT.

*I felt strange at that moment because many mothers are now talking about this test. Doctors should be the ones who know the most updated information in medical science. They should know well about these. But the moment I asked the doctor about the test, it seemed like I introduced the test to the doctor. It was very strange. (31 years old, 1:91*)

Three women from the self-referral group felt that their doctors were unsupportive of their request for DNA-NIPT reporting that they had said that “DNA-NIPT is unnecessary for screening” or “DNA-NIPT is too expensive.” In addition, those who had initiated the thought of testing commented on the lack of follow-up care following DNA-NIPT at the university clinic when they returned to see their own obstetricians. For example, one woman commented:

*I feel that I need to rely on myself whether the test would turn out either positive or negative because my obstetrician didn’t support my decision. I need to find the solutions by myself what to do on the next stage. (38 years old, 1:107*)

#### DNA-NIPT reduces the uncertainty associated with other forms of antenatal testing

Many women said that they experienced some difficulties in interpreting the risk-ratios reported following SU-DSS screening. They described themselves as confused and frustrated by being given a result that consisted of a wide range of probabilities and detection rates.

*After screening, the doctor explained very ambiguously that the risk was about one over a thousand, which is ‘normal’ [i.e., low risk from screening*]*. However, the accuracy was between 80 and 90%. He did not specifically say whether it is 90% or 80%. So, it turned out if it is 80%, there would be 20% of chance of having a baby with Down syndrome. (32 years old, 1:247*)

After screening, women are counseled with the final risk score that is calculated from the combination of factors including age and NT, not by a single factor. However, they did not understand the procedure or said their obstetricians had not explained this clearly.

*I did not know whether the screening was accurate. The doctor told me that I got two problems: one is age, and the other of NT measurement was right on the ‘borderline.’ I did not know what the borderline meant. There was no further explanation. I was not able to understand. (37 years old, 1:240*)

Given the perceived lack of clarity of the SU-DSS results they received before coming to the university clinic, women were of the opinion that this form of screening would not accurately detect aneuploidy because “different doctors would assess risks differently based on subjective assumptions.” When receiving their SU-DSS result, five women reported that they were anxious and had kept asking their doctor for a more definitive answer, which, because of the nature of the test, the obstetricians could not provide. Many women in the study did not understand why their doctor had avoided providing a more accurate risk assessment following SU-DSS and one woman mistakenly speculated that this might be evidence of the practice of defensive medicine.

*No matter who performs the test, women receive 80-90% accuracy. Doctors say that there is no guarantee and it simply is a probability. They keep emphasizing it because they worry about being sued if the baby turns out the other way. (38 years old, 1:170*)

While DNA-NIPT does not fulfill the definition of diagnostic test, as the sensitivity is 99.1%, women described themselves very satisfied with receiving a relatively simple categorical result – “yes or no” – for DNA-NIPT instead of, what they perceived as, the more “*ambiguous*” risk rates they received from SU-DSS.

*DNA-NIPT result is either yes or no. No need for explanation. DNA is like that. By the time I received the result, I was either happy or unhappy. It’s clear… Oh, that 0.9%. I did not worry too much since 99.1% is a high probability. (32 years old, 1:106*)

In contrast to the probability-based result of SU-DSS, women said they did not need a long explanation about their DNA-NIPT result from obstetric professionals. Indeed, receiving a categorical answer about the test result was described as one of the most significant benefits of DNA-NIPT, not least because it is seen as providing a great deal of assurance about the condition of the fetus. 

#### DNA-NIPT provides reassurance about risk and further ‘insurance’

Older women (≥ 35 years) were particularly outspoken about SU-DSS repeatedly commenting on its lack of certainty and the perceived lack of clarity regarding the risk profiles generated by SU-DSS. Many in this group said they were inclined to ignore the results of SU-DSS and assessed their risk solely in relation to their age.

*Over 40 is considered as another line. My birthday is in June and I took screening in July. If I have taken it before June, I would not have gotten this result. Before 40 the risk ratio is about 1/200, but after 40, the risk increases to 1/100 or more. (40 years old, 1:24*)

Another woman who was 34 years old said,

*I am 34 years old. I am not at risk yet. My birthday is December. I will turn into 35 afterwards. Since I am expected to deliver in January next year, I will be 35 years old. Doctors told me that considering the time of my delivery I am considered as high risk.*

While SU-DSS testing result reports combined maternal age with other factors, older women’s motivations for undertaking DNA-NIPT were regarded as straightforward; it was argued that this test enables women, who are already at higher risk because of their age, to avoid the distress caused by being identified as at high-risk by SU-DSS. Thus, DNA-NIPT was seen as providing older women with significant reassurance about what they perceived as age-related risks.

*If I know I am at high-risk because of my age, why don’t I just choose DNA-NIPT? SU-DSS in fact does nothing but to increase more worries. Both DNA-NIPT and conventional SU-DSS have the same procedure and only need drawing the blood. (43 years old, 1:300*)

*Don’t do SU-DSS screening anymore, if you are over 35, which makes mothers be scared all the time. If you are low-risk, that’s fine. But if there is high risk, you will be scared to death. (35 years old, 1:140*)

Women who were not deemed high-risk by SU-DSS, in contrast, although aware that they were not (technically) at high risk based on the screening test results, said they had used DNA-NIPT to confirm their risk status and that they felt more secure when this returned a negative result. For this group DNA-NIPT was, therefore, seen as providing a form of extra insurance.

*There is a long-term consequence, the rest of my life! DNA-NIPT was like “buying insurance” I feel more secure. Use of DNA is better than measure of neck thickness and the table of risk by age. If you are too worried, you can buy your confidence and protection.*

Using the test as insurance meant that women who were not deemed high-risk underwent DNA-NIPT even after receiving a negative result from SU-DSS, and two women went on to have SU-DSS after undergoing DNA-NIPT. This group argued that as long as there is no harm done by testing there was more to be gained from having multiple tests or “*insurance*.” 

#### DNA-NIPT is an easy and safe procedure for women

The view of DNA-NIPT as “a simple blood test” was often expressed by the women we interviewed, who likened DNA-NIPT to the routinized and frequent blood tests they regularly undergo in the first trimester.

*Well, there is nothing unusual about taking blood tests. Every time I visited obstetrics clinic, I was required for blood test, so I didn’t find anything unusual from DNA-NIPT except too much blood drawn for the test.”*

One of the ethical concerns about the implementation of DNA-NIPT is that the relative ease of testing – a straightforward non-invasive blood test – may lead to service providers and users regarding it as an ethically uncontentious procedure[20,30]. The fact that the women in this study generally view DNA-NIPT as “a simple blood test” does highlight that adequate pre-test counseling is important. When women came to the clinic, they said that they remembered receiving a brief explanation of DNA-NIPT, usually given by the nurse, and being given a consent form to sign. 

#### DNA-NIPT is a safe procedure for the fetus

A frequent justification for undertaking prenatal screening is that it supports the reproductive autonomy of pregnant women, allowing them to make choices about whether to continue with an affected pregnancy or opt for TOP. The overwhelming majority of high-risk women in our study said they would consider TOP if the fetus had chromosomal abnormalities.

*If the test came out Down syndrome, I would have aborted it. But, after abortion, my age is no longer young. I will be 42. I am not sure whether I would still have another chance. I would have lost two babies, if it were counted. (42 years old, 1:80*)

Until this was confirmed however, they said that they wanted to minimize risks to their fetus and DNA-NIPT was perceived as enabling them to avoid the risks of miscarriage associated with other testing procedures. Women who had had past experience with IPD or miscarriage stated that they preferred to use DNA-NIPT as it had no procedural risk for the fetus.

*In my last pregnancy, after I took amniocentesis test and I saw blood that night. I was scared. For this time, I have decided to undergo DNA-NIPT. (37 years old, 1:107*)

*I immediately started crying when I heard I was at risk. My doctor told me to calm down since it was not confirmed yet. High risk requires amniocentesis, which carries 1% chance of miscarriage. My husband and I really wanted to have a baby. The last time I was pregnant, I had a miscarriage. This time we were more nervous and cautious. We decided to use DNA-NIPT and not to take the risk. (38 years old, 1:57*)

High-risk women, in particular tended to compare DNA-NIPT with IPD in terms of risk appraisal rather than test accuracy, drawing comparisons between the risks of these procedures, for example, “a fetus may be poked by the needle” and, therefore, potentially harmed in the case of IPD, versus just “blood taking from my arm” in the case of DNA-NIPT. The ease and safety of DNA-NIPT for women was emphasized by drawing comparisons with other invasive procedures such as CVS and amniocentesis, for example, one woman talked about her friend’s description of IPD as a painful procedure.

*My friend told me that a “big” syringe is required to be inserted into the belly. Besides, it may have to repeatedly done a few times. The whole procedure made her scared. A big syringe without anesthesia, she said, was very painful! (39 years old, 1:136*)

The majority of women said they chose DNA-NIPT above more invasive tests because they wanted to eliminate risks to their fetus, and this was justified as also benefiting themselves “what is the best for baby is what is the best for mother.” 

#### DNA-NIPT enables early detection of the fetus’s condition

One frequently voiced ethical justification for the clinical utility of DNA-NIPT is that early detection in pregnancy may offer important benefits for women. A negative test result enables earlier reassurance and, therefore, allows for better opportunities for prenatal bonding. A positive test result offers the opportunity of undergoing selective abortion, which may be physically or psychologically less burdensome when carried out earlier in pregnancy. These ideas were reflected in our interviewees’ narratives. Two women in our study who had a fetus with trisomy 21 in a previous pregnancy undertook DNA-NIPT at 11 weeks, skipping SU-DSS, in order that they might know the condition of the fetus as early as possible. A 40 years old woman who was not found to be high-risk from SU-SSS explained her reasons for accessing DNA-NIPT as follows:

*I aborted my first baby after knowing it has Down syndrome by amniocentesis at 16 weeks. It took 3 weeks for analysis. I received the result at 20 weeks. I thought this is so late because the baby had been kept accompanied with me for 20 weeks and I decided to abort it eventually. My feeling was contradicted and confused. This was my second pregnancy. I knew DNA-NIPT could be done at around 10 weeks of gestation. I wanted to know whether the second baby has Down syndrome as earlier as possible. DNA-NIPT has advantage over amniocentesis. (40 years old, no risk score from SU-DSS*)

In HK SU-DSS can be performed as early as 11 weeks of gestation, and its result is available in a few days; if positive, women can undergo CVS at 11-13 weeks, followed by PCR testing of which result can be reported in one day. Meanwhile, DNA-NIPT takes approximately 2 weeks for reporting; if positive, women would need to undergo a confirmatory test, either CVS or amniocentesis. Thus, for women who are at high-risk, the clinical pathway of confirming aneuploidy through DNA-NIPT is no shorter than conventional procedures. The difference between the two procedures is that the higher accuracy of DNA-NIPT will provide psychological assurance for those have been found to not be at risk after SU-DSS screening. A woman described how her concerns about their pregnancy changed following DNA-NIPT.

*During the first three months of my pregnancy, I was worried that the baby would be lost, so I didn’t dare to tell anyone. I had it at that moment it may be lost in the next moment. It’s like me on a running machine. My heart was racing non-stop. There were so many tests to go through, one after another and so on. I got prenatal depression. I had been thinking a lot what if I could not pass it. After taking DNA-NIPT, I could be sure whether my baby has a problem or not. Now, I have passed it. (42 years old, 1:80*)

#### The perceived cost-benefit ratio of DNA-NIPT

In HK women can choose either privately- or publicly-funded antenatal care. Women from the private sector pay for IPD. In the public sector women may expect IPD to be paid for by government funding as part of their universal routine package of care. However, due to a longer waiting time from SU-DSS to IPD in the public sector, women often decide to directly access the tests in the private sector. Given that many women using the private sector are already paying for antenatal testing, many of our interviewees had explicitly factored cost into their decision-making process. Even though DNA-NIPT is more costly than IPD, given the benefits of clarity, reassurance and insurance associated with the former, the women we interviewed did not perceive that the cost of DNA-NIPT as a barrier to its use, particularly when compared with the costs of other forms of testing (e.g., IPD), other aspects of prenatal care, and delivery.

*Most women give birth in their early 30s. After college, they work for several years, so most likely that they would not have a baby until 30. Since advanced technology could get the screening done with high accuracy, I would think that it is justified to take it. I just need HK$2-3,000 more to assure myself and my baby. (34 years old, 1:138*)

However, while they may not have been worried about the cost of DNA-NIPT, our interviewees also noted the potential for an increase in health inequalities with the introduction of this new technology. As one woman said, “Babies are all the same when they are born. If there is no rich or poor baby at birth, why is this test is only available for rich women?” They suggested, a more equitable approach to the public funding of DNA-NIPT in the future, arguing that the government should subsidize the testing fee for particular groups of women to ensure wider access to the test.

*DNA-NIPT should be more expensive than OSCAR that is about $3,000. Then DNA-NIPT could be $5,000 with the government subsidy of $3,000. After the improvement of the service, it can be offered for free among only women over 35 years old, as OSCAR is free offered by the government for the group of women. (37 years old, 1:29*)

## Discussion

This is the first qualitative study to describe the perceived clinical utility of DNA-NIPT for the detection of trisomy 21 in the fetus among pregnant women where the test is implemented as a service in antenatal screening settings. Whether or not DNA-NIPT should be made available as part of routine antenatal care will depend on a variety of factors not least who will pay for this service. Our study found that DNA-NIPT is currently used by those who can afford to pay as 90% of the women in this study came from higher income groups. However, although socioeconomic status may impact on individuals’ ability to access this technology, and appeared to have some influence on referral patterns, in this instance, it did not affect perceptions of the test’s benefits and the process of testing. Women said they had chosen to undergo DNA-NIPT because it was non-invasive, is easy to use, provides clear categorical results and is less harmful to the fetus. Perceived risk impacts upon women’s perceptions of the utility of DNA-NIPT and their motivations for undergoing this procedure. Women, whether identified as high-risk or not high-risk by SU-DSS, expressed uncertainty and confusion regarding the results of SU-DSS. Women identified to be at high-risk by SU-DSS appeared to regard the DNA-NIPT test as a safe alternative to IPD and to provide clearer indication of their risk status. Women who were not deemed high-risk by SU-DSS saw DNA-NIPT as offering them reassurance about their self-perceived risks as determined by their age or previous obstetric history or as offering additional confirmation that their fetus was not at risk. Our study suggests that women see the risks of aneuploidy as directly related to their age and that consequently, women over 35 years old may expect to have further antenatal confirmatory testing of IPD. These expectations may become increasingly prevalent with the rising age of prime childbearing age in HK, which has reported one of the lowest total fertility rates in the world, at 1.04 per woman [46]. Even those HK women who marry in their 20s commonly delay having a child, thus shortening their childbearing years. Indeed, only 70% of married women now have their first baby during the first 3 years in marriage, with 28.9 as the median age of first marriage [46]. This trend of childbearing at advanced age is seen in other developed countries as well. As many women plan to have only one child at advanced age, it is anticipated that DNA-NIPT may be considered as a preventive measure for high-risk women. These women, they will need to be fully and properly informed about the costs and benefits of DNA-NIPT.

There was evidence that the source of information about DNA-NIPT influenced women’s decisions to access testing. Some women were more knowledgeable about DNA-NIPT than others before their first obstetric visit. These women proactively sought information about DNA-NIPT and tended to be more likely to opt for the test. In fact, this study was conducted during the initial implementation phase of the DNA-NIPT service and captured the earliest adopters of the test. Nonetheless, as with any new technology, a balance will need to be achieved by having the test counselors delivering adequate amount of correct information about the test to the pregnant women. While high-risk women may perceive the use of DNA-NIPT as helping them to avoid what they perceive as risky, invasive procedures, our data suggests that women who were not deemed high-risk perceive DNA-NIPT as a form of extra insurance about their pregnancy. Although the use of DNA-NIPT to provide additional confirmation of other test results may have perceived benefits for individual women, this practice may have wider repercussions. While some may argue that in privately funded healthcare systems women are at liberty to buy “insurance” by taking as many tests as they can afford, this may result in over-testing individuals. More importantly, it can be argued that if women are prepared to pay for this service, then there may be less incentive for governments to incorporate DNA-NIPT into the care pathway, thus impeding the development of cost-effective publicly funded prenatal screening policies. 

### Practice and policy implications

Our study highlights the need to consider how DNA-NIPT can be integrated into first trimester screening and diagnosis. Whether a new test should be added to universal routine care or not needs to be considered from a variety of perspectives. For women, DNA-NIPT offers the opportunity of having a non-invasive test that can give a high-risk result earlier in pregnancy and our interviewees thought this was advantageous. The introduction of new treatments may have implications for providers and professionals as well as patients. Professionals may lack knowledge about certain scientific advances and their impact on public health and this must be addressed. The need to develop expertise in genetic counseling as part of the DNA-NIPT service has been discussed recently [47]. However, the most likely implementation scenario is that we will adapt pre-existing antenatal counseling services to incorporate DNA-NIPT. If this is the case, then the role of nurses and midwives as well as obstetricians in its implementation will be very important. In order to ensure informed consent to undergo DNA-NIPT, patients should be given a clear explanation of the test procedures, including any risks and alternatives and expected outcomes if referral to another site is necessary [30,48]. Providing educational materials in addition to consultations with healthcare providers will help women to be better informed and prepared for undergoing confirmatory IPD if this is needed. In addition, costs and service implications need to be taken into account by service providers. As the test is new the cost benefit literature is limited, however, DNA-NIPT may be cost-effective if it is used by high-risk women between routine SU-DSS and IPD [49,50]. The use of DNA-NIPT as an intermediate test may save other costs associated with invasive procedures and this will impact upon resource allocation and insurance coverage [16]. The cost saving, however, might be dependent upon the provision and extent of screening service prior to DNA-NIPT. Local-specific economic cost-effective analyses should be conducted to calculate the impact of the integration of DNA-NIPT on obstetric care at individual as well as population levels [16,29].

### Limitations and strengths of study

Although women in this study came from across HK, this study was limited to the views and experiences of pregnant women from one obstetrics clinic, and differences may exist for women from other clinics, regions or countries regarding their views of clinical utility, value, and the perceived cost-benefits. In addition, the study was conducted during the early implementation phase of the DNA-NIPT service and captured the views of the early adopters; it is possible that this group may hold particularly negative views of conventional screening procedures. However, using various sampling strategies, the study included a heterogeneous group of women, including women whose monthly income below the median household income of HK and demonstrated the richness of the individual experiences of using DNA-NIPT. Because none of the women who participated in interview received a positive result for aneuploidy, the study provides no information about the views of those who decide to proceed to IPD and go on to terminate/continue with pregnancy. 

## Conclusions

In the future we will need to address broader public health issues such as the changing demography of childbearing and the role that DNA-NIPT might play in this. However, this raises a number of questions, such as: how can we respond to the growing demand for this service amongst older women, how can the service be made available to all and how will a wider uptake of DNA-NIPT impact on healthcare systems, and society more generally? Arguably, quality assured DNA-NIPT based obstetric care can only be scaled up if governments support the adoption of DNA-NIPT as an element of routine antenatal care. One thing that is clear is that we need to develop clear clinical guidelines for the use of DNA-NIPT that address the local context of obstetric healthcare systems, and that health education materials for women who use this technology need to be developed. 
